# Antiproliferative and Proapoptotic Activities of Methanolic Extracts from *Ligustrum vulgare* L. as an Individual Treatment and in Combination with Palladium Complex

**DOI:** 10.3390/ijms13022521

**Published:** 2012-02-22

**Authors:** Milena G. Ćurčić, Milan S. Stanković, Emina M. Mrkalić, Zoran D. Matović, Dragić D. Banković, Danijela M. Cvetković, Dragana S. Đačić, Snežana D. Marković

**Affiliations:** 1 Department of Biology and Ecology, Faculty of Science, University of Kragujevac, Radoja Domanovića 12, 34000 Kragujevac, Republic of Serbia; E-Mails: mstankovic@kg.ac.rs (M.S.S.); c_danijela@yahoo.com (D.M.C.); draganadjacic@kg.ac.rs (D.S.D.); smarkovic@kg.ac.rs (S.D.M.); 2 Department of Chemistry, Faculty of Science, University of Kragujevac, Radoja Domanovića 12, 34000 Kragujevac, Republic of Serbia; E-Mails: emrkalic@kg.ac.rs (E.M.M.); zmatovic@kg.ac.rs (Z.D.M.); 3 Department of Mathematics, Faculty of Science, University of Kragujevac, Radoja Domanovića 12, 34000 Kragujevac, Republic of Serbia; E-Mails: dragic@kg.ac.rs (D.D.B.)

**Keywords:** acridin orange/ethidium bromide assay, antiprolifertative activity, apoptosis, cotreatment, *Ligustrum vulgare* L., MTT assay, palladium, time dependance

## Abstract

The aim of this study is to examine the growth inhibitory effects of methanolic leaf and fruit extracts of *L. vulgare* on HCT-116 cells over different time periods and their synergistic effect with a Pd(apox) complex. The antiproliferative activity of plant extracts alone or in combination with the Pd(apox) complex was determined using MTT cell viability assay, where the IC_50_ value was used as a parameter of cytotoxicity. Results show that antiproliferative effects of *L. vulgare* extracts increase with extension of exposure time, with decreasing IC_50_ values, except for 72 h where the IC_50_ values for methanolic leaf extract were lower than for the fruit extract. The Pd(apox) complex alone had a weak antiproliferative effect, but combination with *L. vulgare* extracts caused stronger effects with lower IC_50_ values than with *L. vulgare* extracts alone. The type of cell death was explored by fluorescence microscopy using the acridin orange/ethidium bromide method. Treatments with plant extracts caused typical apoptotic morphological changes in HCT-116 cells and co-treatments with Pd(apox) complex caused higher levels of apoptotic cells than treatment with plant extracts alone. The results indicate that *L. vulgare* is a considerable source of natural bioactive substances with antiproliferative activity on HCT-116 cells and which have a substantial synergistic effect with the Pd(apox) complex.

## 1. Introduction

Wild privet, *Ligustrum vulgare* L. (Oleaceae) is a semi-evergreen woody branched bush, growing to 5 m tall. The stems are stiff, erect and with grey-brown spotted bark; leaves are lanceolate or broadly elliptical, 2–7 cm long and 0.5–2 cm broad. The flowers are white and fragrant, forming a pyramidal panicle. The fruit is a small glossy black berry of 6–8 mm diameter, containing one to four seeds. It usually inhabits forest ecosystems of Central and Southern Europe, North Africa and Southwest Asia, and is often cultivated as an ornamental plant [1].

Leaves of *L. vulgare* are used for disease prevention or treatment in folk medicine in southern Europe [2], mostly because of their immunomodulatory [3], cardioprotective [4], antibacterial [5], antidiabetic effects [6], and it has also been found that different leaf and fruit extracts of this plant can scavenge OH and DPPH radicals [7]. From the traditional uses and later scientific findings, which suggested cytotoxic activity against HeLa cells [8], *L. vulgare* may be a potential candidate as an antiproliferative agent against cancer cell lines. Different *in vitro* assays performed in *L. vulgare* leaves show broad pharmacological potential of this plant due to the presence of flavonoids, phenylpropanoids and terpenoids (mainly secoiridoids) [2,9]. However, there is no data available on the antiproliferative activity of *L. vulgare* fruit extracts.

Nature has long been an important source of medicinal agents. An impressive number of modern drugs have been isolated or derived from natural sources, based on their use in traditional medicine [10,11]. Ethnopharmacological or traditional use of plants often results in the discovery of new biologically active molecules [12]. A wide variety of secondary metabolites obtained from plants are tested for their ability to treat cancer. Various plant-derived anti-cancer drugs are known to be effective against proliferating cells. In addition, there are chemotherapeutic compounds commonly used to fight cancer, such as cisplatin [13]. Although cisplatin is a valuable antitumor drug, it has several disadvantages including side effects such as nephrotoxicity, ototoxicity, neurotoxicity, myelotoxicity, peripheral neuropathy and hematological toxicity [14]. The adverse effects observed in patients receiving cisplatin chemotherapy have generated new areas of research mainly focused on the search for new nonplatinum metal-based complexes displaying low toxicity with improved therapeutic properties. A new [Pd(apox)] complex was synthesized in an attempt to obtain compounds with superior chemotherapeutic index in terms of increased bioavailability, higher cytotoxicity, and lower side effects than cisplatin. The coordination chemistry of palladium(II) is very similar to that of platinum(II), but the higher lability in ligand exchange at the Pd centre (10^5^-fold *vs*. Pt) may cause rapid hydrolysis processes leading to the dissociation of the complex and formation of very reactive species unable to reach their biological targets. This issue could be overcome by using bulky heterocyclic and chelating ligands. Here we used the chelating ligand H_2_apox(*N*,*N*′-*bis*(3- aminopropyl)oxamido) to synthesize a neutral *N*,*N*′-*bis*(3-aminopropyl)oxamido palladium(II) complex. In the equatorial plane, the complex contains fully deprotonated bidentate ligands capable of forming a five-membered (oxamido) backbone.

The aim of this study is to examine the growth inhibitory effects of methanolic leaf and fruit extracts of *L. vulgare*, on human colon cancer cells (HCT-116) over different time periods by two different assays along with their synergistic effect with Pd(apox) complex, as well as to examine the type of cell death.

## 2. Results and Discussion

### 2.1. Time- and Dose-Dependent Effects of *L. vulgare* Extracts on Cell Proliferation

**Figure 1:**
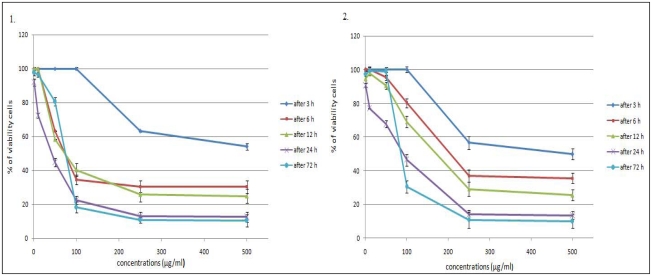
Effects of methanolic leaf extract (**1**) and fruit extract (**2**) of *L. vulgare* on HCT-116 cell proliferation. The cells were treated with various concentrations of extracts and antiproliferative activities were measured by MTT assay after 3, 6, 12, 24 and 72 h of treatment. Results were expressed as means ± SE for three independent determinations.

We conducted two *in vitro* experiments on human colon cancer cells—HCT-116 cell line. The MTT cell viability assay *in vitro* is the primary stage for screening. Cells were treated with different concentrations of plant extracts (in concentration range from 1 to 500 μg/mL) at different duration ranges (3, 6, 12, 24, 48 or 72 h) to clarify the role of the time of exposure. Figure 1 shows the dose and time-dependent activity of extracts for all duration ranges. We chose different incubation times to determine the most effective one. While the inhibition of cell viability was obtained after 3 h of treatment only for the highest concentrations, prolonged treatments (6, 12, 24 and 72 h) caused significant inhibition of viability for all concentrations. The anti-proliferative activity of the *L. vulgare* extracts depended on the exposure time. However, the cell proliferation at 72 h treatment resulted in an increase in IC_50_ values (inhibitory dose inhibited cell growth by 50%). A significant time-dependent inhibition of cell proliferation was not obtained when only exposing the cells to the lower concentrations after 72 h, and this did not strengthen along with the longer time of exposure. Low concentrations can kill cells immediately after treatment, but after longer time of exposure, cells recover and they can stimulate some proliferative effects in surviving cells. This correlates with the previously obtained results with HCT-116 cell line [15].

Table 1 presents *in vitro* cytotoxic activities of the methanolic extracts of *L. vulgare*, which were expressed by IC_50_ values. The IC_50_ value was used as a parameter for cytotoxicity. The results showed that maximum effect has methanolic leaf extract after 24 h (28.2 μg/mL), indicating this time as the most effective time exposure for antiproliferative effects.

**Figure 2:**
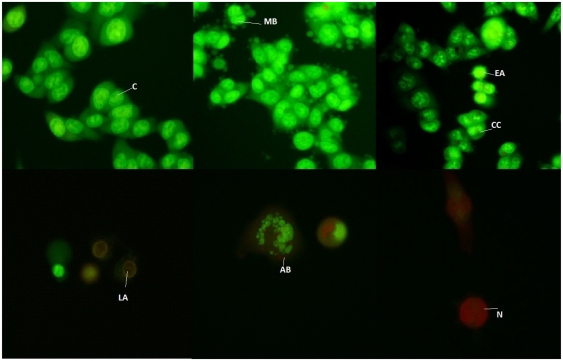
Typical morphological changes of HCT-116 cells induced by 100 μg/mL methanolic extracts of *L. vulgare*, stained with AO/EB. The images were taken using fluorescence microscopy at 400×. C: control, no treated cells; MB: membrane blebbing; CC: chromatin condensation; EA: early apoptosis; LA: late apoptosis; AB: apoptotic body; N: necrosis.

Natural bioactive substances can modify redox status and interfere with basic cellular functions (cell cycle, apoptosis, inflammation, angiogenesis, invasion and metastasis) [16]. Apoptosis is important in embryological development, cell proliferation, cell differentiation, elimination of seriously damaged cells or tumor cells by chemopreventive or chemotherapeutic agents and many other physiological processes [17]. Apoptotic cells and bodies are rapidly recognized by macrophages before cell lysis, and then can be removed without inducing inflammation. Therefore, the induction of apoptosis is an important mechanism of chemoprevention and chemotherapy of cancer. To determine whether the inhibition of cell proliferation by methanolic extracts from *L. vulgare* was due to the induction of apoptosis, we assessed the latter with the acridine orange/ethidium bromide method. Proapoptotic activity of methanolic extracts from *L. vulgare* was investigated with respect to the morphological shape of cells by fluorescence microscopy. Fluorescence microscopy images clearly showed morphological changes such as reduction in size and cell volume, cell shrinkage, membrane blebbing, chromatin condensation, nuclear fragmentation and formation of apoptotic bodies of treated cells (Figure 2).

Table 2 summarizes the results obtained with AO/EB double staining. The percentages of viable, apoptotic and necrotic cells were noted for different incubation periods with extracts (Table 1). Typical morphological changes after different time periods are shown on Figure 2. A time dependent increase in induction of apoptosis was also observed, except for 72 h treatment, which was also apparent in the results from the MTT assay. Compared with spontaneous apoptosis observed in control cells, HCT-116 treated with 100 μg/mL methanolic extracts of *L. vulgare* showed increased percentages of early apoptotic cells (the higest increase showed methanolic leaf extract of *L. vulgare*: 45.7%, after 24 h), and increased percentages of necrotic cells (the highest increase showed methanolic fruit extract of *L. vulgare*: 3.1%, after 72 h).

The AO/EB and the MTT assays showed similar time-dependent effects on viability but the percent of viabilities were different. In MTT assays absorbance of control cells were considered as 100% and percentages of viable cells were calculated as ratio to the control, while in AO/EB assay percentages of viable cells are real numbers, calculated as ratio to the total number of cells in treatment. If there is a difference in viability it is because mitochondrial function assays (MTT) detected cell death earlier than others, while apoptosis indicating assays (AO/EB) detected cell death later in the process, which is in correlation with other data [18].

According to obtained results we can conclude that *L. vulgare* has antiproliferative properties which increase with exposure time up to 24 h, when extracts have the best activity. The methanolic leaf extract has better antiproliferative activity than the methanolic fruit extract with lower IC_50_ values, and induces a higher percentage of apoptotic cells after 12 h of exposure. Extracts were able to induce cell apoptosis, but they did not induce necrosis in HCT-116 cells. Necrotic cells appeared in a very small percentage.

The mechanism of action is unclear and, possibly, multiple compounds in the herbal extracts are involved [19]. Plant-derived chemical constituents, flavonoids, iridoids, coumarins, phenyl propanes and essential oil were reported to be the main constituents in *L. vulgare* but which are responsible for biological activities is not known [20].

### 2.2. Antiproliferative Effects of *L. vulgare* Extracts in co-Treatment with Pd(apox) Complex

Cancer is the third leading cause of death worldwide, preceded by cardiovascular and infectious diseases. There are standard chemotherapeutic drugs for treatment of cancers. Several studies have shown that chemotherapeutic drugs have harmful effects on health and can lead to the development of drug resistance in tumor cells, which limit the clinical success of cancer chemotherapy [21,22]. Recent reports show that chemotherapeutic drugs and natural compounds with known anti-cancer activity could be used in combination therapy to reduce the systemic toxicity of chemotherapeutic agents [23,24]. So-called natural compounds with anti-cancer activity often modify many intracellular signaling pathways simultaneously. Previous reports demonstrated that many side effects of common used chemotherapy agents are consequences of induction of oxidative stress, which could be palliated by antioxidant food and plants [25].

In this study, the antiproliferative and proapoptotic activity of the methanolic extracts of *L. vulgare* and the chemotherapeutic drug—Pd(apox) complex were investigated in HCT-116 cells by MTT cell viability assay and AO/EB assay. Different combinations of Pd(apox) and plant extracts were used to find those combinations showing the highest potential to reduce viability of HCT-116 cells. Combination assays were performed using appropriate concentrations of methanolic extracts of *L. vulgare* (1, 50, 100 and 250 μg/mL) with appropriate concentrations of Pd(apox) complex (100 and 250 μM). The Pd(apox) complex was added after 3, 6 and 24 h of cell incubation with plant extract. Cell viability was measured after 24 h (for cells where Pd(apox) was added after 3 and 6 h of cell exposure to plant extracts) and after 72 h (for cells where Pd(apox) was added after 24 h of cell exposure to plant extracts). Cells treated with the same final concentrations of the extracts or chemotherapeutic drugs alone were also examined.

The MTT assay indicated that the Pd(apox) complex had very weak antiproliferative activity (96.7% and 94.3% of viability cells in treatment with 100 μM and 250 μM Pd(apox) for 24 h; 96.1% and 91.3% of viability cells in treatment with 100 μM and 250 μM Pd(apox) for 72 h). Literature data also imply that palladium compounds are not stable and therefore in the cellular environment will be translabilized easily by other biomolecules such as GSH, giving intermediates [26].

**Figure 3:**
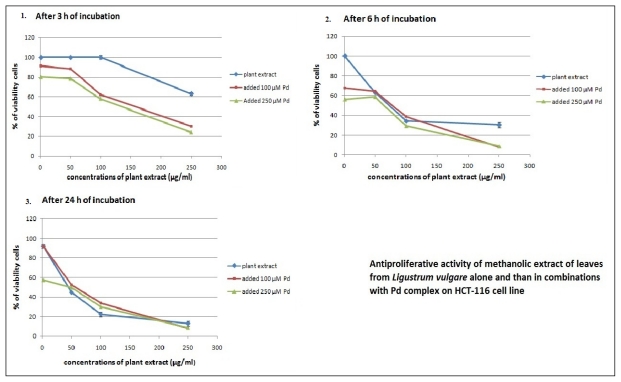
Antiproliferative activity of methanolic leaf extract of *L. vulgare* alone and in combinations with Pd(apox) complex on HCT-116 cell line. Cells were treated with plant extracts and after 3, 6 and 24 h of incubation Pd(apox) complex was added. The antiproliferative activity was measured by MTT assay after 24 h (1 and 2) and 72 h (3) of initial treatment with plant extracts. Results were expressed as means ± SE for three independent determinations.

**Figure 4:**
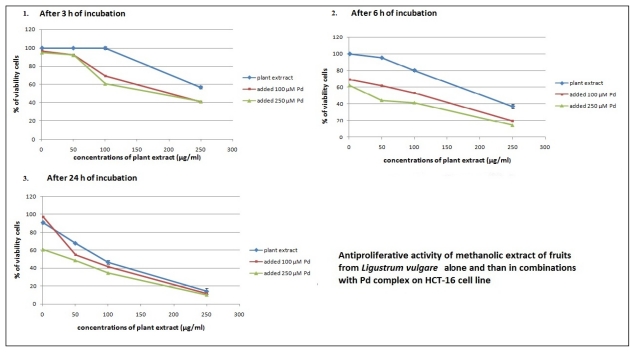
Antiproliferative activity of methanolic fruit extract of *L. vulgare* alone and in combinations with Pd(apox) complex on HCT-116 cell line. Cells were treated with plant extracts and after 3, 6 and 24 h of incubation Pd(apox) complex was added. The antiproliferative activity was measured by MTT assay after 24 h (1 and 2) and 72 h (3) of initial treatment with plant extracts. Results were expressed as means ± SE for three independent determinations.

*L. vulgare* extracts were selectively toxic against HCT-116 cell line over different time periods, and in combination with Pd(apox) complex produced an increased growth inhibitory effect (Figures 3 and 4) with lower IC_50_ values (Table 3). The addition of Pd(apox) complex significantly reduces the IC_50_ values (calculated in relation to the concentration of plant extract), although Pd (in individual treatment) did not show antiproliferative activity. The best antiproliferative activity was observed with the combination of leaf extract and 250 μM Pd(apox) complex measured after 72 h (IC_50_ = 4.8 ± 0.6). The results indicate that Pd(apox) complex and plant extracts have synergistic effects on cell proliferation inhibition.

Similar viability results for Pd(apox) complex were obtained from the AO/EB assay (96.9% and 94.9% of viability cells in treatment with 100 μM and 250 μM Pd(apox) for 24 h; 96.7% and 92.7% of viability cells in treatment with 100 μM and 250 μM Pd(apox) for 72 h). Table 4 summarizes the percentages of viable, apoptotic and necrotic cells observed by addition of the Pd(apox) complex in combination with plant extracts over different time periods. A time-dependent increase in induction of apoptosis was also observed. The HCT-116 treated with different drug combinations showed an increase in the percentage of early apoptotic cells (combination of methanolic leaf extract of *L. vulgare* and 250 μM Pd(apox) complex: 45.2%, after 6 h showed the higest increase of early apoptotic cells), and increased the percentage of late apoptotic cells (the highest increase showed with the combination of methanolic leaf extract of *L. vulgare* and 250 μM Pd(apox) complex, after 72 h).

It might be possible that *L. vulgare* extracts could induce reactive oxygen species (ROS) in the induction of apoptosis. The ROS production were confirmed for some components from *L. vulgare* such as flavonoids, terpenoids and other components [27,28]. These findings suggest that *L. vulgare* extracts could induce apoptosis in HCT-116 cells may be by a ROS mediated mechanism and inhibit the glutathione conjugate export pump. Also the extracts could increase the sensitivity of HCT-116 cells to Pd(apox) complex. As a result, the sensitivity of tumor cells to anticancer drugs was increased [29,30]. The explanation of all the aspects and mechanism of the synergistic effects of *L. vulgare* extracts and Pd(apox) complex will be the next step in our investigation of this problem.

## 3. Experimental Section

### 3.1. Chemicals

Methanol was purchased from “Zorka pharma”, Serbia. Dublecco’s Modified Eagle Medium (DMEM) was obtained from GIBCO, Invitrogen, USA. Fetal bovine serum (FBS) and trypsin-EDTA were from PAA (The cell culture company), Austria. Acridine orange was obtained from Acros organic, New Jersey, USA. Dimethyl sulfoxide (DMSO), ethidium bromide and 3-[4,5-dimethylthiazol-2-yl]-2,5- diphenyltetrazolium bromide (MTT) were obtained from SERVA, Germany. The ligand *N*,*N*′-*bis*(3- aminopropy1)oxamide(H^2^apox) was synthesized by following the procedures described in the literature. Potassium tetrachloro-paladate(II) was purchased from Aldrich and used as supplied. Reagent grade, commercially available, chemicals were used without further purification.

### 3.2. Preparation of [Pd(apox)]

Of the ligand H^2^apox(*N,N*′-*bis*(3-aminopropyl)oxamide) [31] 0.303 g was dissolved in 5 cm^3^ of water and then 0.163 g of K_2_PdCI_4_ was added slowly to the stirred solution. The pH of solution in the course of the addition of K_2_PdCI_4_ was kept approximately neutral by addition of KOH solution. The resulting solution was filtered and then allowed to stand at room temperature. Yellow crystals were obtained. Analytical calculation for C_8_H_16_N_4_O_2_Pd: C, 31.33; H, 5.26; N, 18.27. Found: C, 31.02; H, 5.33; N, 17.98.

### 3.3. Preparation of Plant Extracts

Prepared plant material (10 g) was transferred to dark-coloured flasks. It was soaked in 200 mL of methanol and stored at room temperature. After 24 h, the infusions were filtered through Whatman No. 1 filter paper and the residue was re-extracted with equal volume of solvents. After 48 h, the process was repeated. Combined supernatants were evaporated to dryness under vacuum at 40 °C using a Rotary evaporator. The obtained extracts were kept in sterile sample tubes and stored in a refrigerator at 4 °C.

### 3.4. Cell Preparation, Culturing and Treatments

HCT-116 cell line was obtained from American Type Culture Collection. Cells were maintained in DMEM supplemented with 10% Fetal Bovine Serum, with 100 units/mL penicillin and 100 μg/mL streptomycin. Cells were cultured in a humidified atmosphere with 5% CO_2_ at 37 °C. Cells were grown in 75 cm^2^ culture bottles supplied with 15 mL DMEM, and after a few passages, cells were seeded in a 96-well plate. The experiments were done at 70 to 80% confluence.

HCT-116 cells were seeded in a 96-well plate (10,000 cells/well). After 24 h of cells incubation, the medium was replaced with 100 μL medium containing methanolic leaf and fruit extracts of *L. vulgare* at different concentrations (1, 10, 50, 100, 250 and 500 μg/mL). Untreated cells served as controls. For determination of time dependence, cell viability was determined by MTT assay and type of cell death was determined by AO/EB assay under a fluorescent microscope after appropriate incubation times with plant extracts (3, 6, 12, 24 and 72 h).

For investigation of combination effects of plant extracts and Pd(apox) complex on cell proliferation, 10,000 cells/well were seeded in 96-well plates. After 24 h the cells were exposed to four concentrations of methanolic leaf and fruit extracts of *L. vulgare* (1, 50, 100 and 250 μg/mL). Pd(apox) complex (100 and 250 μM) was added after 3, 6 and 24 h of cell incubation with plant extracts. Absorbance was measured and cells were counted after 24 h (for cells where Pd(apox) was added after 3 and 6 h of cell exposure to plant extracts) and 72 h (for cells where Pd(apox) was added after 24 h of cell exposure to plant extracts) from initial treatments with plants. AO/EB assay for analysis of cell death was also used over the same time intervals and the same combinations of concentrations. Cells were also treated with Pd(apox) complex (100 and 250 μM) alone.

### 3.5. Cell Viability Assay (MTT Assay)

The cell viability was determined by MTT assay [32]. The proliferation test is based on the color reaction of mitochondrial dehydrogenase in living cells by MTT. At the end of the treatment period, MTT (final concentration 5 mg/mL PBS) was added to each well, which was then incubated at 37 °C in 5% CO_2_ for 2–4 h. The colored crystals of produced formazan were dissolved in 150 μL DMSO. The absorbance was measured at 570 nm on a Microplate Reader. Cell proliferation was calculated as the ratio of absorbance of the treated group divided by the absorbance of the control group, multiplied by 100 to give a percentage proliferation.

### 3.6. Fluorescence Microscopic Analysis of Cell Death

We used acridine orange/ethidium bromide (AO/EB) double staining assay [33]. Acridine orange is taken up by both viable and nonviable cells and emits green fluorescence if interrelated into double stranded nucleic acid (DNA) or red fluorescence if bound to single stranded nucleic acid (RNA). Ethidium bromide is taken up only by nonviable cells and emits red fluorescence by intercalation into DNA. We distinguished four types of cells according to the fluorescence emission and the morphological aspect of chromatin condensation in the stained nuclei. Viable cells have uniform bright green nuclei with an organized structure. Early apoptotic cells (which still have intact membranes but have started to undergo DNA cleavage) have green nuclei, but perinuclear chromatin condensation is visible as bright green patches or fragments. Late apoptotic cells have orange to red nuclei with condensed or fragmented chromatin. Necrotic cells have uniformly orange to red nuclei with a condensed structure. The amount of 20 μL of dye mixture (10 μL/mg AO and 10 μL/mg EB in distilled water) was mixed with 100 μL cell suspension (10,000 cells/mL) in a 96-well plate. After the incubation times with the drugs the suspension was immediately examined and viewed under a Nikon inverted fluorescent microscope (Ti-Eclipse) at 400× magnification. We observed untreated cells as controls. A minimum of 300 cells was counted in each sample. Results were expressed as means ± SE for three independent determinations.

### 3.7. Statistical Analysis

The data are expressed as means ± standard errors (SE). Biological activity was examined in three individual experiments, performed in triplicate for each dose. Statistical significance was determined using the Student’s *t*-test or the one-way ANOVA test for multiple comparisons. A *p* value <0.05 was considered as significant. The magnitude of correlation between variables was done using a SPSS (Chicago, IL) statistical software package (SPSS for Windows, ver. 17, 2008). The IC_50_ values were calculated from the dose curves by a computer program (CalcuSyn).

## 4. Conclusions

The study concludes that *L. vulgare* might be considered as a potential source of metabolites which could be developed as precursors for anti-cancer drugs. There is/are active constituent(s) in *L. vulgare* with anti-proliferative effect against HCT-116 cells. It is nessesary in the future to determine the type of constituents with potential activities. Isolation, purification and consideration of possible applications of these active compounds are in prospect.

To summarize, our results demonstrate that methanolic leaf and fruit extracts of *L. vulgare* had the best antiproliferative and proapototic effects after 24 h with prolonged exposure being less effective. The extracts from leaves had higher antiproliferative activity (with lower IC_50_ values) than fruit extracts. Combinations of methanolic extracts of *L. vulgare* with Pd(apox) complex in HCT-116 cell lines had better antiproliferative effects than either agent alone, with significantly lower IC_50_ values. Leaf extracts had better synergistic effects with the Pd(apox) complex than fruit extracts. Further studies are needed to assess the underlying mechanisms (e.g., signal transduction pathways) leading to growth inhibition induced by single agents and combinations both *in vitro* and *in vivo*. A positive outcome of such studies would increase the efficacy of existing chemotherapies with reduced toxicity to normal tissues in the treatment of human colon cancer.

## Figures and Tables

**Table 1:** Growth inhibitory effects—IC_50_ values (μg/mL) of methanolic leaf and fruit extracts of *L. vulgare* on HCT-116 cell line after 3, 6, 12, 24 and 72 h of treatment.

Methanolic extracts of *L. vulgare*	After 3 h	After 6 h	After 12 h	After 24 h	After 72 h
leaf	> 500	159.5 ± 1.92	147.8 ± 7.29	28.2 ± 0.76	64.6 ± 3.84
fruit	> 500	325.9 ± 4.51	240.8 ± 3.64	47.4 ± 3.54	108.0 ± 5.32

**Table 2:** Different values of viable, apoptotic and necrotic cells as percentage of all cells, measured by AO/EB fluorescence staining, after treatment by 100 μg/mL of methanolic extracts of *L. vulgare*. The percentages of cells were measured after 3, 6, 12, 24 and 72 h of treatment. VC-viable cells; EA-early apoptosis; LA-late apoptosis; N-necrosis.

		VC	EA	LA	N
3 h	untreated cells	96.5 ± 0.19	3.4 ± 0.26	0.1 ± 0.13	
	leaf extract	83.3 ± 1.37	16.4 ± 1.34	0.2 ± 0.09	0.1 ± 0.08
	fruit extract	80.0 ± 4.14	16.1 ± 4.48	3.1 ± 0.23	0.7 ± 0.1
6 h	untreated cells	87.3 ± 7.09	12.7 ± 0.28	0.1 ± 0.105	−
	leaf extract	74.4 ± 3.53	25.2 ± 3.59	0.4 ± 0.027	−
	fruit extract	77.0 ± 6.11	22.9 ± 6.10	−	−
12 h	untreated cells	90.9 ± 0.08	8.7 ± 0.215	0.4 ± 0.29	−
	leaf extract	56.1 ± 2.08	41.3 ± 2.48	2.5 ± 1.57	0.1 ± 0.07
	fruit extract	73.6 ± 4.78	25.9 ± 4.71	0.2 ± 0.17	0.1 ± 0.06
24 h	untreated cells	96.3 ± 0.38	2.2 ± 0.10	1.4 ± 0.29	−
	leaf extract	52.5 ± 2.10	45.7 ± 2.47	1.9 ± 0.36	−
	fruit extract	53.2 ± 3.85	45.6 ± 4.52	1.2 ±0.66	−
72 h	untreated cells	97.1 ± 3.58	2.9 ± 2.15	−	−
	leaf extract	68.3 ± 2.59	29.4 ± 1.87	2.3 ± 0.35	−
	fruit extract	71.9 ± 1.13	21.8 ± 0.98	1.2 ± 1.01	3.1 ± 0.15

**Table 3:** Growth inhibitory effects—IC_50_ values (μg/mL) of methanolic leaf and fruit extracts of *L. vulgare* in combination with Pd(apox) complex on HCT-116 cell line. IC_50_ values was calculated in relation to the concentration of plant extract. Cells were treated with plant extracts and after 3, 6 and 24 h of incubation Pd(apox) complex was added. The antiproliferative activities were measured by MTT assay after 24 and 72 h of initial treatment with plant extracts. Results were expressed as means ± SE for three independent determinations.

	Measured after 24 h		Measured after 72 h
	
combination of drugs	Pd(apox) complex added after 3 h of cell incubation with plant extract	Pd(apox) complex added after 6 h of cell incubation with plant extract	Pd(apox) complex added after 24 h of cell incubation with plant extract
Leaf extract and 100 μM Pd	269.5 ± 2.89	13.8 ± 1.21	27.1 ± 1.47
Leaf extract and 250 μM Pd	121.1 ± 3.25	5.5 ± 0.75	4.8 ± 0.65
Fruit extract and 100 μM Pd	347.9 ± 4.63	33.5 ± 1.56	145.4 ± 1.26
Fruit extract and 250 μM Pd	422.2 ± 5.41	8.1 ± 1.01	6.0 ± 0.25

**Table 4:** Different values of viable, apoptotic and necrotic cells as percentage of all cells measured by AO/EB fluorescence staining. Cells were treated with 100 μg/mL plant extracts and after 3, 6 and 24 h of incubation Pd(apox) complex was added. The cell percentages were measured after 24 and 72 h of initial treatment with plant extracts. Results were expressed as means ± SE for three independent determinations. VC-viable cells; EA-early apoptosis; LA-late apoptosis; N-necrosis.

Time of cell counting			VC	EA	LA	N
after 24 h		control, untreated cells	96.3 ± 0.38	2.2 ± 0.10	1.4 ± 0.29	-
after 72 h			97.1 ± 3.58	2.9 ± 2.15	-	-
24 h after treatment		100 μM Pd(apox) complex	98.1 ± 0.89	1.9 ± 0.28	-	-
		250 μM Pd(apox) complex	96.6 ± 3.81	3.4 ± 1.09	-	-

**Pd(apox) complex**		**combination of drugs**				

24 h after initial treatment with plant extracts	after 3 h	leaf extract and 100 μM Pd	74.1 ± 0.93	25.7 ± 0.47	0.2 ± 0.29	-
		leaf extract and 250 μM Pd	62.3 ± 6.35	36.5 ± 4.39	1.1 ± 0.39	-
		fruit extract and 100 μM Pd	79.1 ± 0.77	20.7 ± 1.24	0.2 ± 0.74	-
		fruit extract and 250 μM Pd	68.9 ± 4.05	31.1 ± 1.87	-	-
	after 6 h	leaf extract and 100 μM Pd	44.9 ± 4.76	41.1 ± 0.79	13.7 ± 1.25	0.2 ± 2.56
		leaf extract and 250 μM Pd	32.1 ± 2.68	45.2 ± 5.05	20.8 ± 1.74	1.8 ± 3.21
		fruit extract and 100 μM Pd	59.1 ± 1.57	32.7 ± 0.41	8.2 ± 1.13	-
		fruit extract and 250 μM Pd	50.1 ± 2.91	39.7 ± 2.31	10.1 ± 2.01	-
72 h after initial treatment with plant extracts	after 24 h	leaf extract and 100 μM Pd	37.1 ± 1.58	26.7 ± 1.87	34.3 ± 1.36	1.8 ± 0.97
		leaf extract and 250 μM Pd	30.5 ± 2.02	29.5 ± 2.14	37.9 ± 1.91	2.1 ± 1.67
		fruit extract and 100 μM Pd	40.8 ± 2.14	30.6 ± 1.57	25.9 ± 1.36	2.6 ± 1.07
		fruit extract and 250 μM Pd	42.9 ± 1.64	31.3 ± 1.42	24.8 ± 1.42	1.0 ± 1.34
